# Horizontal and Vertical Transmission of a Mycovirus Closely Related to the Partitivirus RhsV717 That Confers Hypovirulence in *Rhizoctonia solani*

**DOI:** 10.3390/v15102088

**Published:** 2023-10-13

**Authors:** Aili Sun, Lianjing Zhao, Yang Sun, Yingrui Chen, Chengyun Li, Wenhan Dong, Genhua Yang

**Affiliations:** 1 State Key Laboratory for Protection and Utilization of Bio-Resources in Yunnan, Yunnan Agricultural University, Kunming 650201, China; sunaili@xtbg.ac.cn (A.S.); zhao12345620220714@163.com (L.Z.); yang_sun@126.com (Y.S.); 15368745562@163.com (Y.C.); licheng_yun@163.com (C.L.); 2 CAS Key Laboratory of Tropical Forest Ecology, Xishuangbanna Tropical Botanical Garden, Chinese Academy of Sciences, Kunming 650000, China

**Keywords:** RsPV-BS5, sexual spores, transmission, virus particles, reduce pathogenicity, transcriptome

## Abstract

Rhizoctonia solani virus717 (RhsV717) was isolated from the *Rhizoctonia solani* (*R. solani*) AG-2 strain Rhs717. This study isolated a virus designated as Rhizoctonia solani partitivirus BS-5 (RsPV-BS5) from the *R. solani* AG-3 strain BS-5, the causal agent of tobacco target spot disease. The virus was identified as a strain of RhsV717. Transmission electron microscopy (TEM) images showed that RsPV-BS5 had virus particles with a diameter of approximately 40 nm. Importantly, it can be horizontally transmitted through hyphal anastomosis and vertically transmitted via sexual basidiospores. Furthermore, this study demonstrated that RsPV-BS5 infection significantly impedes mycelial growth and induces hypovirulence in tobacco leaves. Thus, RsPV-BS5 presents a promising avenue for biocontrolling tobacco target spot disease. Transcriptome analysis unveiled differential expression of four genes related to cell wall-degrading enzymes between two isogenic strains, 06-2-15V and 06-2-15. These findings shed light on the molecular mechanism through which RsPV-BS5 reduces host pathogenicity.

## 1. Introduction

*Rhizoctonia solani* (*R. solani*) is an important soilborne necrotrophic fungal pathogen, which consists of 14 subgroups (AG1–AG14), based on the hyphal anastomosis reaction between *R. solani* strains [1,2]. *R. solani* AG-3 mainly infects potato, tobacco, and tomato. *R. solani* isolated from potatoes are commonly referred to as AG-3 PT to distinguish them from AG-3 isolates pathogenic to tobacco (AG-3 TB) and tomato (AG-3 TM), which can be differentiated based on variations in ITS sequences [3,4,5]. Tobacco (*Nicotiana tabacum* L.) is an important economic crop around the world. In recent years, tobacco target spot disease, mainly caused by *R. solani* anastomosis group 3 (AG-3), has been widely found in many countries [6]. For instance, occurrences of tobacco target spots have been documented in the United States, South Africa, Italy, and Bulgaria, leading to severe losses of over 80% in some cases [7]. This disease was first diagnosed in China in 2006 and has had a considerable adverse impact on tobacco yield and quality [8,9]. Consequently, there is an urgent need to develop innovative methods for controlling tobacco target spot disease, with phytopathogen biocontrol emerging as one of the most effective and environmentally friendly approaches [10].

Mycoviruses are prevalent among major fungi. The first mycovirus was discovered in the edible mushroom *Agaricus bisporus* (phylum: Basidiomycota) in 1962 [11,12]. Subsequently, mycoviruses have been identified in various significant fungal taxa, including Basidiomycota, Ascomycota, Chytridiomycota, Deuteromycota, and Zygomycota [13]. Advancements in next-generation sequencing have led to the documentation of more than 100 mycoviruses in *R. solani* [14,15], primarily falling within families such as *Barnaviridae*, *Botourmiaviridae*, *Deltaflexiviridae*, *Endornaviridae*, *Hypoviridae*, *Fusariviridae*, *Megabirnaviridae*, *Mitoviridae*, *Partitiviridae*, and unclassified groups [16]. *Partitiviridae* is a branch of these 22 taxa. All the viruses in this family are spherical with a size of 34–42 nm and have double-stranded RNA (dsRNA) genomes [17]. The viruses of this family are divided into five genera, namely, *Alphapartitivirus*, *Betapartitivirus*, *Gammapartitivirus*, *Deltapartitivirus*, and *Cryspovirus* [18,19,20]. While most mycoviruses tend to establish latent infections, some can induce hypovirulence in their host fungi, rendering them potential agents for biocontrol against plant fungal diseases [21,22,23]. Rhizoctonia solani virus717 (RshV717) was isolated from the *R. solani* AG-2 strain Rhs717 with a dsRNA, which was weakly virulent on cabbage and radish and moderately virulent on snapbean and maize [24,25]. The successful utilization of hypoviruses to control chestnut blight (*Cryphonectria parasitica*) in Europe has spurred the exploration of mycoviruses with the potential to induce hypovirulence in their plant pathogenic hosts, thereby reducing their pathogenic impact [26]. Notable examples included the ssDNA mycovirus Sclerotinia sclerotiorum hypovirulence-associated DNA virus 1 (SsHADV-1) [22], Rhizoctonia solani partitivirus 2 (RsPV2) infecting *R. solani* AG-1 IA [27], Sclerotinia sclerotiorum partitivirus 1 (SsPV1/WF-1) isolated from *S. sclerotiorum*, which induces hypovirulence in its host [28], and Rhizoctonia solani dsRNA virus 5 (RsRV5) isolated from AG-1 IA, which also contributes to decreased host pathogenicity [29], among others. 

In host fungi, partitiviruses spread horizontally, mainly through mycelial anastomosis, but also spread vertically through gamete and sporogenesis [30]. Asexual spores usually have a higher probability of transmission, but the type of virus and host commonly affect the transmission efficiency. For example, Cryphonectria hypovirus 1 (CHV1) can be 100% transmitted to conidiospore offspring, but only 50% of the offspring of sexual spores are virulent [31]. *R. solani* does not produce any asexual spores. Nevertheless, the sexual stage (teleomorph) is also hard to induce in vitro [32,33]. However, the vertical transmission of fungal viruses isolated from the *R. solani* AG-3 pathogen has not been reported until now.

In this study, we obtained and characterized Rhizoctonia solani partitivirus BS-5 (RsPV-BS5) from the *R. solani* AG-3 strain BS-5. Phylogenetic analyses revealed that the RsPV-BS5 is similar to RhsV717; both are members of the *Betapartitivirus* in *Partitiviridae.* We obtained a RsPV-BS5-cured strain from the *R. solani* AG-3 strain BS-5 (referred to here as YBS-5) and compared it with the RsPV-BS5-infected original strain BS-5 in terms of mycelial growth and pathogenicity to the host. Furthermore, we conducted horizontal and vertical transmission studies of RsPV-BS5, and transcriptome technology was used to explore the molecular mechanism of the interaction between RsPV-BS5 and *R. solani* AG-3.

The objective of this study was to isolate the molecular and biological characteristics of the RsPV-BS5 virus from *R. solani* and to provide a theoretical basis and data support for the role of RsPV-BS5 as a resource for the biological control of tobacco target spot disease.

## 2. Materials and Methods

### 2.1. Extraction and Detection of dsRNA

Strain BS-5, belonging to *R. solani* AG-3 TB, was isolated from tobacco leaves. Strain BS-5 was cultured on potato dextrose agar plates for two days. Then, we collected the mycelia and transferred them to potato dextrose broth at 28 °C with orbital shaking at 100 rpm for one week for the subsequent extraction of double-stranded RNA (dsRNA) and total RNA [34,35]. The dsRNA of strain BS-5 was extracted using the CF-11 cellulose (Sigma-Aldrich, Louis, MO, USA). For purification, dsRNA suspensions were treated with DNase I and S1 nuclease (TaKaRa, Dalian, China) to remove genomic DNA and ssRNA, respectively [36]. Total RNA was extracted using an RNA Easy Fast Plant Tissue Kit (Tiangen, Beijing, China) and was used as a template to synthesize first-strand cDNA.

### 2.2. cDNA Synthesis, Molecular Cloning, and Sequencing

In addition, total RNA was extracted from the mycelia of the *R. solani* strain BS-5 and used for Illumina NovaSeq 6000 platform sequencing to produce viral contigs (Majorbio, Shanghai, China). The raw data were processed, merged and compared on the National Center for Biotechnology Information (NCBI) website to determine the viral genome sequence [37]. A 25 µL PCR reaction mixture was prepared, containing components like 12.5 µL ExTaq Mix (TaKaRa, Dalian, China), 1 µL cDNA template, 1 µL specific primers (Supplementary Table S1), and 9.5 µL dd H_2_O [38,39]. The resulting PCR products were analyzed using 1% agarose gel electrophoresis, and the presence of the virus was confirmed by visualizing the stained gel. The purified PCR amplicons were inserted into the pMD18-T vector and then transformed into *Escherichia coli* DH5α competent cells. Three positive clones were selected from the transformed competent cells for further analysis (Sangon, Shanghai, China). To obtain the sequences at the 5′ and 3′ ends of the putative mycoviruses, we used the RNA-ligase-mediated rapid amplification of cDNA end (RLM-RACE) techniques [40,41]. An adaptor (PC3-T7 loop primer) (Supplementary Table S1) was ligated to the purified dsRNA with T4 RNA ligase at 4 °C for 16 h. After purification, cDNA synthesis was performed to create a complementary DNA [35]. The 5′ and 3′ terminal sequences were obtained using the primer PC2 and specific primers (Supplementary Table S1). The resulting PCR products were purified, cloned and sequenced.

### 2.3. Sequence Analysis

For open reading frame (ORF) determination, we used the NCBI ORF Finder program to locate regions that could encode proteins (http://www.ncbi.nlm.nih.gov/gorf/gorf.html (accessed on 10 April 2022). After determining the deduced amino acid sequences from the ORFs, a BLASTp search was performed using the NCBI database to compare the protein sequences and identify similar sequences and conserved domains. Motif searches were performed in the CDD database (http://www.ncbi.nlm.nih.gov/Structure/cdd/wrpsb.cgi (accessed on 20 April 2022). A phylogenetic tree was constructed based on the deduced amino acid sequences of the RdRp regions. We used the Molecular Evolutionary Genetics Analysis (MEGA version 7.0, Mega Limited, Auckland, New Zealand) software version 7.0 with the maximum-likelihood (ML) method and with 1000 bootstrap replicates in this analysis.

### 2.4. Virus Particle Extraction and Detection

The virus particles were purified using the method described by Sanderlin and Ghabrial with minor modifications [42]. The extraction of virus particles was carried out using a sucrose density gradient method. BS-5 was selected and cultured in a potato glucose liquid medium for eight days. Approximately 10 g of mycelium was selected and dried with sterile absorbent paper and 0.05 mmol/L of phosphate-buffered saline (PBS buffer, PH 7.4). The powder was ground with liquid nitrogen and placed in a 50 mL centrifuge tube on ice for high-speed centrifugation at 3500 r/min for 15 min. The Supernatant was taken, supplemented with 12 mL chloroform, and centrifuged at 12,000 r/min at 4 °C for 10 min at a high speed. The supernatant containing virus particles was taken; then, after 2 h at 4 °C and 30,000 r/min, the supernatant was discarded, the precipitate was collected, and 1 mL 0.05 M PBS (PH 7.4) was added to dissolve the precipitate to obtain the crude extract. The crude extract was centrifuged at 60,000 r/min for 2 h through 10–60% sucrose gradient centrifugation, and the virus particles were distributed at 20–30%. After careful collection, the solution was dissolved in 200 μL of 0.05 M PBS. In preparation for visualization, the solution containing virions was diluted fivefold using a 0.05 M sodium phosphate solution. Subsequently, copper mesh was employed for adsorption for 5 min, followed by negative staining with 2% (*w*/*v*) phosphotungstic acid (PTA) for an additional 5 min. The virus particles were then observed using a transmission electron microscope (TEM, Tecnai G2).

### 2.5. Horizontal and Vertical Transmission of RsPV-BS5

Strain 06-2-15, belonging to *R. solani* AG-3, was isolated from tomato leaves. Notably, this strain did not harbor any mycovirus. To assess the potential for the horizontal transmission of RsPV-BS5 from an infected donor strain (BS-5) to a recipient strain (06-2-15), co-cultivation experiments were conducted. BS-5 and recipient 06-2-15 strains were co-cultivated on potato dextrose agar (PDA) at 28 °C over three days. The strain 06-2-15 was sensitive to tomato leaves, and tomato leaves were selected for pathogenicity testing. During the eco-cultivation experiments, we obtained the newly infected RsPV-BS5 strain 06-2-15V. Then, the mycelia of strains BS-5, 06-2-15 and 06-2-15V were enlarged and cultured in a liquid medium containing potato glucose. After that, we detected the dsRNA and extracted the total RNA using RT-PCR (Supplementary Table S2). For the vertical transmission experiment, sporulation was induced by introducing the *R. solani* AG-3 strain BS-5 into barley grains at the base of the tomato stems. The basidiospores resulting from this process were cultured on PDA for three days and then transferred to a liquid medium containing potato glucose for further growth. After this, dsRNA was detected, and total RNA was extracted through RT-PCR. Two specific primers were designed and synthesized to facilitate these experiments based on RdRp and CP sequences (as indicated in Supplementary Table S2). There were three biological and three technical replicates in the transmission experiments. The statistical analysis was performed using a Student’s *t* test. Values were considered significant if the *p*-value was 0.05 or less.

### 2.6. Virus Curing

To cure the RsPV-BS5-infected *R. solani* AG-3 strain BS-5, the sample was cultured on PDA supplemented with 100 µg/mL ribavirin and incubated at 32 °C for three days in darkness. We removed a mycelium disk with a diameter of 6.0 mm from the ribavirin-treated PDA plate. We inoculated this mycelium disk onto a new agar plate that contained no nutrients at 32 °C in the dark for three days. After incubation, we cut a hyphal tip with a diameter of 2–3 mm from the growing edge of the agar plate and then transferred it to a new PDA plate supplemented with ribavirin. We repeated this process of transferring hyphal tips to new plates successively until the virus was eliminated. The RsPV-BS5 was finally removed and named strain YBS-5.

### 2.7. Determination of Growth Rate

We determined and compared the growth rates of the virus-infected strain BS-5 and the virus-cured strain YBS-5 on PDA. Then, we obtained mycelial blocks with a diameter of 6.0 mm from the virus-infected strain BS-5 and the virus-cured strain YBS-5 using a puncher followed by culture at 28 °C. The center of the mycelial block was chosen as the cross point to draw a vertical line. The growth length of the mycelium was recorded every day until the colony diameters covered more than 90% of the plate. Then, the average growth speed of the mycelia was calculated. All measurements of the growth rate on the culture plates were performed with at least three biological replicates (samples from different fungal cultures) and three technical replicates.

### 2.8. Virulence Assay

Virulence assays of both *R. solani* strains BS-5 and YBS-5 were conducted on tobacco leaves (*Nicotiana tabacum* L.). Here, strains BS-5 and YBS-5 were initially cultured on PDA, and the mycelium plugs of each were inoculated on the tobacco leaves and incubated in a growth chamber at 28 °C with 90% relative humidity. The lesion sizes were measured up to 5 days post-inoculation (dpi). All inoculations were repeated at least three times.

### 2.9. RNA-Seq and Data Analysis

The strain 06-2-15 and its virus-infected strain 06-2-15V were cultured on PDA plates covered with cellophane membrane in an incubator at 28 °C for seven days. Fungal mycelia were collected to isolate total RNA using a Tiangen polysaccharide polyphenol Kit (TIANGEN, Beijing, China). For RNA-Seq library preparation, we used 5 µL of total RNA per sample. The RNA quality and integrity were determined using an Agilent 2100 Bioanalyzer (Agilent Technologies Co., Ltd., Palo Alto, CA, USA) and 1% agarose gel electrophoresis. To generate Illumina cDNA libraries, we used the NEBNext^®^ Ultra™ Directional RNA Library Prep Kit for Illumina (New England Biolabs, Ipswich, MA, USA). We conducted RNA deep sequencing on the Illumina NovaSeq 6000 platform (Novogene, Beijing, China). We performed initial quality control of the sequencing data using CASAVA software (version 1.8.2, Illumina, San Diego, CA, USA), which included filtering out the reads with adapters containing undetermined bases (N) and low-quality reads. At the same time, the Q20, Q30 and GC contents were calculated for the clean data. The clean reads of the *R. solani* AG-3 strain 06-2-15V and 06-2-15 build an index of the reference genome using HISAT2 (version 2.0.5, CCB at JHU, Baltimore, MD, USA) to pair the end clean reads with reference to the genome (https://ftp.ncbi.nlm.nih.gov/genomes/all/GCA/000/524/645/GCA_000524645.1_Rhizoctonia_solani_AG-3/ (accessed on 10 July 2022). We calculated the gene expression levels using DESeq2 software (version 1.20.0, University of North Carolina, Raleigh, NC, USA) and identified differentially expressed genes (DEGs) using DESeq2, with genes having an adjusted *p*-value ≤ 0.05 considered as differentially expressed. The GO enrichment analysis and statistical enrichment of differentially expressed genes in the KEGG pathway were performed using cluster profiler software (version 3.8.1, Southern Medical University, Guangzhou, China).

## 3. Results

### 3.1. Nucleotide Sequences of RsPV-BS5 Genomic dsRNAs

**Figure 1:**
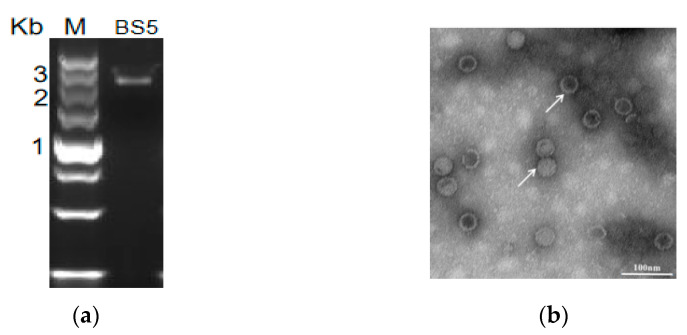

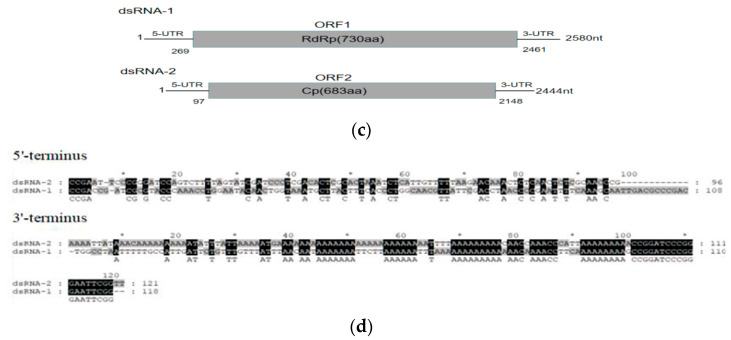
The characteristics of RsPV-BS5. (**a**) Electrophoresis analysis of nucleic acid samples treated with enzyme on 1% agarose gel, nucleic acid samples treated with S1 nuclease and DNase I, M indicates molecular markers; (**b**) RsPV-BS5 particles from strain BS-5, arrows point to the virion form. TEM images (negative staining) of the virus particles of RsPV-BS5. (**c**) Genomic organizations of dsRNA-1 and dsRNA-2 of a dsRNA mycovirus RsPV-BS5 in *R. solani.* (**d**) Terminal sequence domains of RsPV-BS5 genome. Identical sequences of the 5′-UTR and 3′-UTR of the two dsRNAs are reverse highlighted. An asterisk (*) indicates a distance of 10 bases from the previous number.

Because the two dsRNAs were similar, electrophoresis detected that the size of dsRNA fragments was approximately 2.5 kbp (Figure 1a). The virus particle of RsPV-BS5 was spherical with a diameter of approximately 40 nm (Figure 1b). There was one dsRNA-1 (RdRp) with a total length of 2580 nt (GenBankOK392630), 5′-UTR length of 269 bp and 3′-UTR length of 119 bp and that contained a large read frame (ORF1) with a RdRp sequence of 730 aa. For another dsRNA-2 (CP), the full length was 2444 nt (GenBankOK392631), the 5′-UTR length was 96 bp, and the 3′-UTR is 296 bp long. It contained a large reading frame (ORF2) with a coding region ranging from 97 bp to 2148 bp (Figure 1c). As shown in Figure 1, a complete ORF-encoding CP sequence containing 683 aa was predicted. Adenine-rich sequences were shared between the 3′ terminal sequences of the discontinuous polyA tail dsRNA fragment (Figure 1d).

### 3.2. Amino Acid Sequence and Phylogenetic Analysis of Partitivirus RsPV-BS5

**Figure 2:**
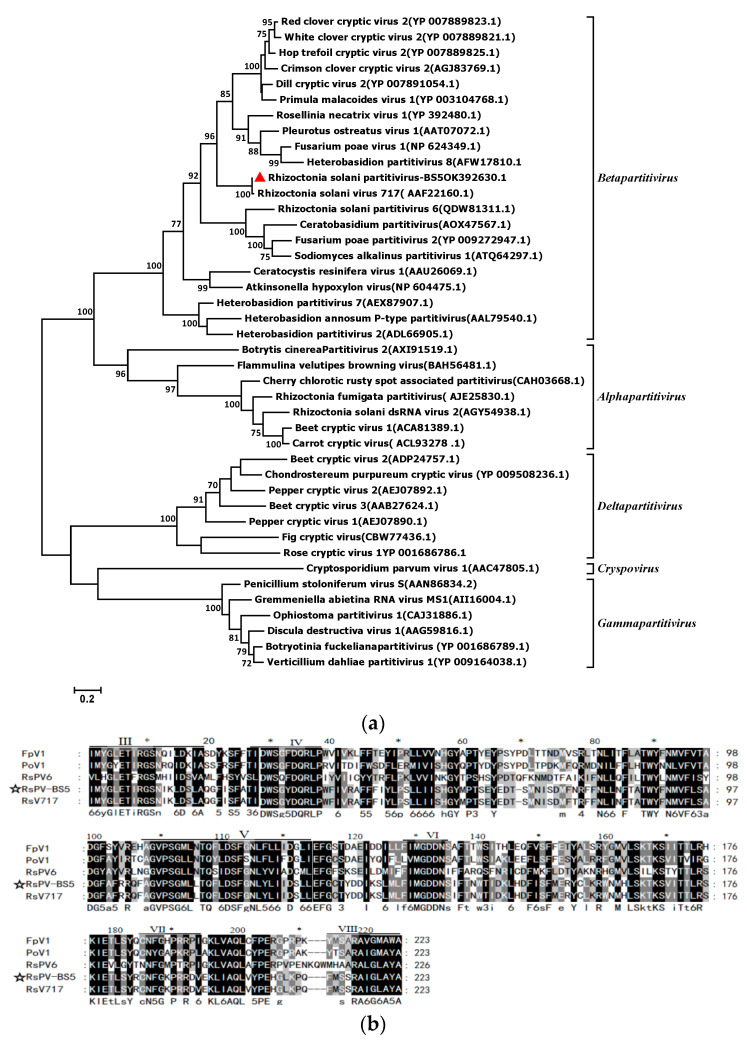
(**a**) Phylogenetic tree based on aligning the amino acid sequences of the RdRp of RsPV-BS5 and other selected viruses of the family *Partitiviridae.* The tree was constructed by the maximum-likelihood method in MEGA 7 with 1000 bootstrap replicates. The mycoviral sequences in the phylogenetic tree are indicated by virus names followed by GenBank accession numbers. A red triangle showed the RsPV-BS5 in this study. (**b**) Multiple alignments of RsPV-BS5 RdRp domains with those of selected mycoviruses of *Betapartitivirus.* Number (I–Ⅷ) represented the eight conserved motifs characteristic of RdRps. An asterisk (*) indicates a distance of 10 bases from the previous number.

To analyze the relationships between RsPV-BS5 and other mycoviruses, the maximum-likelihood method with 1000 bootstrap replicates was used based on the RdRp sequences of RsPV-BS5 and 34 other selected partitiviruses (Figure 2a). The phylogenetic tree of RdRp encoded by each virus showed that RsPV-BS5 was closest to RhsV-717 (Supplementary Table S3). Therefore, the genome organization, amino acid sequence alignments and phylogenetic analyses indicated that RsPV-BS5 belonged to *Betapartivirus*. Furthermore, the fact that we found six conserved motifs (III to VIII) within the RdRp domain suggests that these regions of the protein sequence are highly conserved across different members of the family *Partitiviridae* (Figure 2b).

### 3.3. RsPV-BS5 Can Horizontally Transfer to the Virus-Free Strain 06-2-15

**Figure 3:**
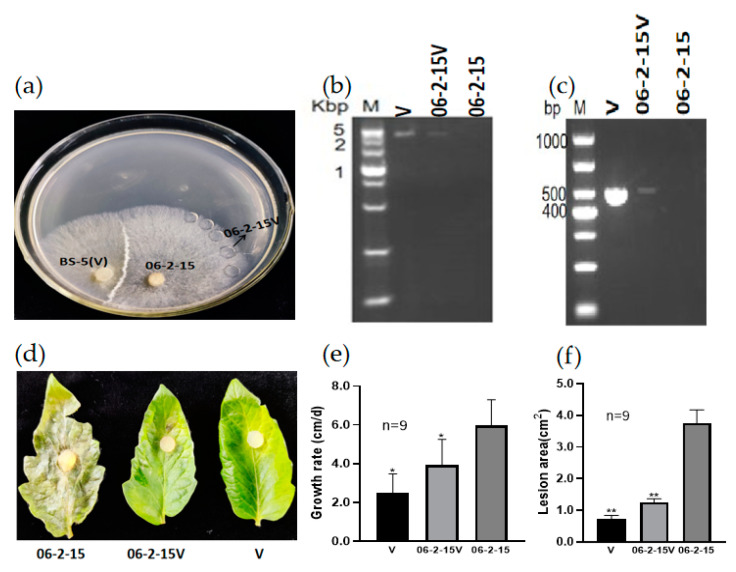
The horizontal transmission results of RsPV-BS5. (**a**) donor strain BS-5 and recipient strain 06-2-15 cultures on PDA plates at three days; (**b**) dsRNA extraction results; (**c**) RT-PCR detection results; (**d**) Pathogenicity. The symptoms on detached tomato leaves caused by strains 06-2-15, 06-2-15V, BS-5(V) incubated at 28 °C for 72 h; (**e**) Growth rate of donor strain BS-5(V), recipient strain 06-2-15 and newly infected RsPV-BS5 strain 06-2-15V on PDA plates ; (**f**) Lesion area of donor strain BS-5(V), recipient strain 06-2-15 and newly infected RsPV-BS5 strain 06-2-15V. Results in each histogram shown in panels (**e**,**f**) were expressed as arithmetic means ± standard errors of the means. One asterisk (*) indicates a significant difference (*p* < 0.05), and two asterisks (**) indicate a highly significant difference (*p* < 0.01) among strains of *R. solani* according to the Student’s *t* test.

To investigate whether RsPV-BS5 could be introduced into the virus-free strain 06-2-15, the horizontal transmission potential of RsPV-BS5 was trialed in a co-culture with a donor strain BS-5 and recipient strain 06-2-15 cultures for three days, through which the newly infected RsPV-BS5 strain 06-2-15V was obtained (Figure 3a). DsRNA was detected, and total RNA was extracted for the RT-PCR detection of RsPV-BS5 using specific primers (64F2/R2) (Supplementary Table S2). The results showed that RsPV-BS5 could spread horizontally through mycelium anastomosis (Figure 3b,c). After that, the isolated tomato leaves were inoculated with the virus-free strain 06-2-15, the virus-carrying strain 06-2-15V and the virus-carrying strain BS-5 (denoted by V), which were placed on the surface of the tomato leaves for 3–5 days, and the area of disease spots was measured. In addition, the effect of RsPV-BS5 on fungal virulence was evaluated based on the sizes of the lesions on the tomato leaves caused by the three strains 06-2-15, 06-2-15V and V (Figure 3d). Three days after inoculation, the average lesion areas caused by 06-2-15V and V were smaller than those caused by 06-2-15 (Figure 3e,f), indicating that RsPV-BS5 induced hypovirulence in the horizontal transmission strain 06-2-15V. This was highly significant according to Student’s *t* test. Values were considered significant if *p* < 0.01.

### 3.4. Vertical Transmission of RsPV-BS5

**Figure 4:**
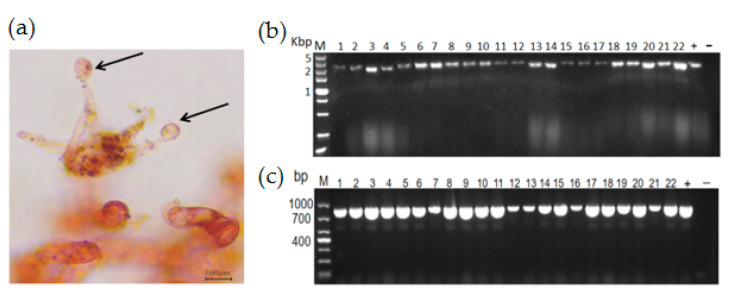
Results related to vertical transmission. (**a**) Basiodiospores were incubated on concave slides; the arrows point to the basiodiospores. (**b**) DsRNA was treated with S1 nuclease and DNase I, 1–22: single basidiospore isolation offsprings culture strains dsRNA results, +: strain BS-5 M: molecular marker, Kbp: 1000 base pairing; (**c**) 1–22: basidiospore culture strains RT-PCR results, +: strain BS-5, −: negative control.

The vertical transmission of RsPV-BS5 occurred mainly through sexual spores (Figure 4a). In this study, we obtained 22 single-basiciospore isolation offspring, from which we extracted dsRNA and total RNA. Therefore, we performed RT-PCR detection with specific primers (BS5CP-F/R) (Supplementary Table S2). The dsRNA and PCR amplicons of the 22 single-basiciospore isolation offsprings were subjected to running electrophoresis (Figure 4b,c). The results showed that the RsPV-BS5 could statically exist in the sexual spores of *R. solani* AG-3.

### 3.5. RsPV-BS5 Curing

**Figure 5:**
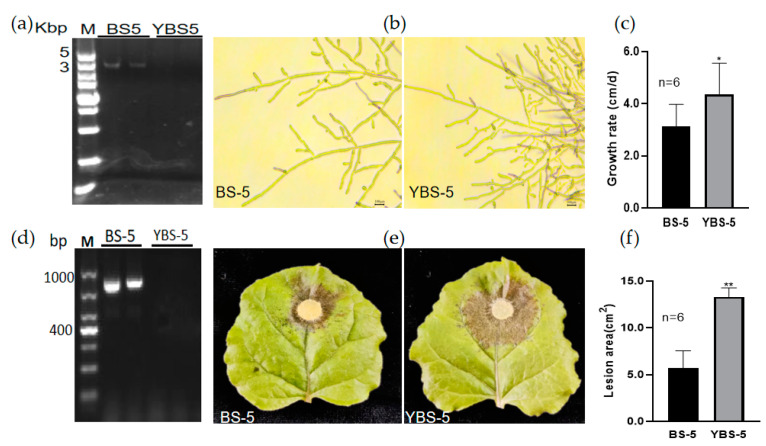
Hypovirulence-associated traits of strain BS-5 of *R. solani*. (**a**)The dsRNA segments detecting in strains BS-5 and YBS-5; (**b**) Microscopic observation of the colony margin in strains BS-5 and YBS-5. (**c**) Comparison of average mycelial growth rates on PDA plates; (**d**) The result of RT-PCR in strains BS-5 and YBS-5; (**e**) Pathogenicity. The symptoms on detached tobacco leaves caused by strains BS-5 (**left**) and YBS-5 (**right**) incubated at 28 °C for 72 h. (**f**) Comparison of average lesion areas caused by BS-5 and YBS-5. Results in the histograms shown in panels (**c**,**f**) were expressed as arithmetic means standard errors of the means. One asterisk (*) indicates a significant difference (*p* < 0.05), and two asterisks (**) indicate a highly significant difference (*p* < 0.01) among strains of *R. solani* according to the Student’s *t* test.

To determine the effect of the virus RsPV-BS5 on the fungal host, we tried to cure the RsPV-BS5-infected *R. solani* AG-3 strain BS-5, as described in Section 2, and a derivative virus-transfected strain YBS-5 was finally obtained. The mycelial morphologies of these two strains, BS-5 and YBS-5, were compared under the same conditions. The dsRNA segments present in strain BS-5 were detected, but we could not detect any in the mycelia of YBS-5 (Figure 5a). The results showed that YBS-5 was thicker than BS-5 (Figure 5b). RsPV-BS5 infection reduces mycelial growth (Figure 5c). The segment in strain BS-5 was confirmed as the genome of RsPV-BS5 through RT-PCR with the RsPV-BS5-specific primer (BS5CP-F/R) (Supplementary Table S2) using total RNA as the templates; however, it was not detected in strain YBS-5 (Figure 5d). In addition, the effect of RsPV-BS5 on fungal virulence was evaluated based on the size of the lesions on tobacco leaves caused by the two strains, BS-5 and YBS-5 (Figure 5e). After three days of inoculation, the average lesion areas caused by BS-5 were smaller than those caused by YBS-5 (Figure 5f), indicating that RsPV-BS5 induced hypovirulence.

### 3.6. RNA-Seq Analysis of R. solani AG-3 PT Response to RsPV-BS5 Infection

We conducted an RNA-seq experiment comparing the expression of fungal host genes in the *R. solani* AG-3 PT strains 06-2-15V and 06-2-15 in response to RsPV-BS5 infection. Our data analysis showed that for the samples of strains 06-2-15V and 06-2-15, there were a total of 44 million and 43 million reads, respectively, with an average of 86.01% and 86.18% reads, respectively, which were aligned to the *R. solani* AG-3 (Supplementary Table S4). The Gene Ontology (GO) (Figure S1) and Kyoto Encyclopedia of Genes and Genomes (KEGG) pathway enrichment analysis (Figure S2) results are outlined in Supplementary File S1.

**Figure 6:**
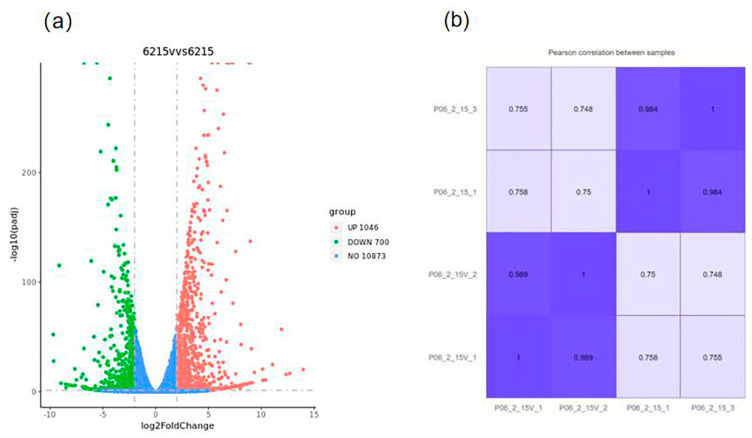
Differentially expressed genes between two isogenic strains, 06-2-15V and 06-2-15 of *R. solani* AG-3, using the transcriptomic technique. (**a**) Volcano plot was a commonly used visualization tool in transcriptomic analysis to represent the significance (usually −log10(FDR)) of differential gene expression on the *Y*-axis and the magnitude of the fold change (logFC) on the *X*-axis, which indicated the extent of the difference in gene expression between the two samples; (**b**) Correlation check of samples 06-2-15V and 06-2-15. R^2^ is the square of the Pearson correlation coefficient.

**Figure 7:**
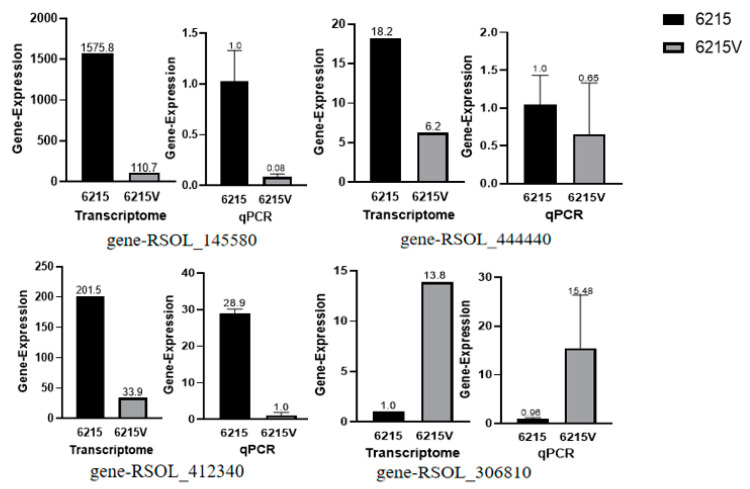
Expression of four genes related to cell wall degrading enzymes. Validation of RNA-sequencing data obtained for *R. solani* AG-3 strain 06-2-15V and 06-2-15 using reverse transcription-quantitative PCR analysis. The expression of each gene on the left is the FPKM value of transcriptome sequencing, and the qPCR result on the right. Results in the histograms shown in panels expressed significant differences among strains of 06-2-15 and 06-2-15V in *R. solani* according to the Student’s *t* test.

In this study, we used absolute logFC ≥ 2 and *p*-value ≤ 0.05 to define DEGs. A total of 1746 differential genes were obtained, of which 1046 were upregulated, and 700 were down-regulated. The “volcano plot” showed the distribution of the DEGs between strains 06-2-15V and 06-2-15 (Figure 6a). A correlation check of RNA-seq revealed similar expression patterns and R^2^ > 0.75 among the three biological repetitions of *R. solani* (Figure 6b). Compared to the gene expression data of strain 06-2-15, a total of four genes (gene-ROSL_145580, gene-ROSL_444440, gene-ROSL_412310 and gene-ROSL_306810) related to cell-wall-degrading enzymes in which the expression was altered were found in the RsPV-BS5-infected strain 06-2-15V, with three upregulated genes (gene-ROSL_145580, gene-ROSL_444440 and gene-ROSL_412310) and one down-regulated gene (gene-ROSL_306810). Gene-ROSL_145580 and gene-ROSL_444440 were supposed to encode a polygalacturonase (PG) domain-containing protein. Gene-ROSL_412310 was predicted to encode a pectin methylesterase (PE) protein. Gene-ROSL_306810 was predicted to encode β-1, 4-endoglucanase (Cx) (Figure 7).

## 4. Discussion

In the scope of our current study, we have provided comprehensive molecular evidence that the virus isolates originating from the *R. solani* AG-3 strain BS-5 constitute a novel strain of the previously described RhsV717. This new strain was designated as RsPV-BS5. Here, we have reported the complete genomic sequence, genome organization, and various biological attributes of the fungal virus RsPV-BS5. Previous research showed that partitiviruses have two essential dsRNA genome fragments, dsRNA-1 and dsRNA-2, each 1300–2500 bp in length [30]. In our study, the genome sequence of RsPV-BS5 also includes two essential dsRNA fragments, namely dsRNA-1 (2580 bp) and dsRNA-2 (2444 bp). For most mycoviruses, the 5′ terminal sequence is conserved between different genome segments; they are involved in the transcription, replication, and packaging of viral RNA [43]. Recently, some studies demonstrated that the 5′ and 3′ terminal sequences of mycoviruses isolated from *R. solani* AG-3 were not conserved in all seven dsRNAs [44]. Similarly, in our study, it was also found that the 5′ terminal of the dsRNA-1, 2 of RsPV-BS5 was not highly conserved. It was not determined whether they were all isolated from the host *R. solani* AG-3, but they shared a conserved 5′ terminal “CCGA”. Adenine-rich sequences are shared between the 3′ terminal sequences of the two dsRNA fragments, but some disconnection occurs in the middle. “Interrupted” polyA tails have also been reported in other members of the *Partitiviridae* family, with interrupted polyA tails involved in the replication of the virus [45]. Our study found that RsPV-BS5 significantly “interrupted” the polyA tails at the 3′ terminals. This has been reported for other members of the *Partitiviridae*, where poly-A tail interruption was detected at the 3′ ends of the dsRNA-1 and dsRNA-2 of RhsV717 virus [25]; for example, Pleustreatus virus 1 (PoV1) is a dimorphic virus that has an “interrupted” poly-A tail with a 3′ terminal sequence [45]. In the case of *R. solani*, it has been found that the AG-1 IA anastomosis group that attenuated the fungal virus RsRV5 had poly-A tail interruption at the 3′ end [30]. Our phylogenetic analyses showed that RsPV-BS5 had a high similarity with the reported partitivirus RhsV717. RsPV-BS5 was identified as a new strain of RhsV717.

Horizontal transmission was mainly carried out through the hyphae fusion of the same anastomosis group [30]. This method has been used in previous studies to conduct relevant studies on RsPV6, RsPV7 and RsPV8 [39]. To clarify the transmission of RsPV-BS5, we successfully introduced the RsPV-BS5 into the healthy virus-free strain 06-2-15 by means of the hyphae fusion technique.

In addition, this study provides the first evidence of the vertical transmission of RsPV-BS5 through sexual spores in *R. solani*. *R. solani* AG-3 does not produce any asexual spores, and it is difficult to induce sexual spores in vitro [29]. Different fungal viruses spread with different levels of efficiency in spores [46]. Vertical transmission is carried out mainly through asexual and sexual spores produced by fungi, passed from parent to offspring [47]. CHV1 has been reported to be 100% transitable to conidiospore offspring but only 50% virulent in the offspring of sexual spores [32]. Wang et al. reported that the vertical transmission rate of Fusarium graminearum hypovirus 1 through asexual spores reached 100% [48]. It has been reported that the average carrier rate of the basophore virus, a dsRNA virus, the pathogen of *Agaricus bisporus* “La France Disease”, is 65% to 75% [49]. The dsRNA mycovirus isolated from *Fusarium graminearum* did not affect colony morphology and was transmissible through conidia and ascospore with an incidence of 30-100% [50]. Lentinula edodes mycovirus HKB (Lev-HKB) was carried in more than 90% of the progenies of Lev-HKB-infected *Lentinus edodes* [51]. There are more than 100 fungal viruses found in *R. solani* [15], but there are few studies on the vertical transmission of mycoviruses. Fortunately, we obtained sexual spores of *R. solani* AG-3. It was found that the obtained basidiospores carried RsPV-BS5, which proved that RsPV-BS5 can carry out the vertical transmission of *R. solani*. This was the first report indicating that a fungal virus is transmitted by sexual spores in *R. solani*.

To obtain virus-cured strains of fungi, a common practice in mycovirology research is to investigate the effects of mycovirus infections on their fungal hosts. Various methods can achieve this, like cycloheximide treatment, ribavirin treatment, hyphal tip isolation, protoplast regeneration, single-spore hybridization, heat treatment, or frozen and lyophilized treatments [52,53,54,55]. Previously, the *R. solani* strain YNBB-111 was used for protoplast regeneration and ribavirin treatment to eliminate the mycovirus, but no virus-cured strain was obtained [44]. Our study successfully obtained a virus-free strain by cutting hyphal tips combined with a high-temperature treatment method in our laboratory. Compared with the RsPV-BS5-cured strain YBS-5, the RsPV-BS5-infected strain BS-5 exhibited the following differences: (a) inhibited mycelial growth and (b) a reduced the area of the lesions on the tobacco leaves. There are many reports showing that partitiviruses reduce host pathogenicity, such as Heterobasidion partitivirus 13 [19] or Sclerotinia sclerotiorum partitivirus 1 [26], which cause hypovirulence or are associated with the growth alteration of their host. Bhatti found that the presence of a gammapartitivirus in *Aspergillus fumigatus* caused significant reductions in radial growth and biomass [56]; Zheng et al. described an alphapartitivirus causing hypovirulence in *R. solani* [27]. Interestingly, Sasaki et al. showed that a megabirnavirus of the root rot pathogen *Rosellinia necatrix* confers hypovirulence with the aid of a co-infecting partitivirus [57]. In addition, Botryosphaeria dothidea chrysovirus 1 (BdCV1) and Botryosphaeria dothidea partitivirus 1 (BdPV1) are co-infecting *Botryosphaeria dothide*, BdCV1 being associated with the hypovirulence of a phytopathogenic fungus [58]. Horizontal transmission and RsPV-BS5 curing experiments proved that RsPV-BS5 could cause hypovirulence in *R. solani*. This useful technique might provide the possibility of biocontroling tobacco target spot disease.

After RsPV-BS5 infection, the number of differentially expressed upregulated genes was usually higher than that of down-regulated genes, which may be related to the expression of low-virulent fungal virus infection. CHV1 infection affected 1023 differentially expressed genes (DEGs), of which 753 were upregulated, and 270 were down-regulated [59]. In a study by Ding et al., 187 genes were screened for differential expression before and after infection when the virions of SsHADV-1 infected rape leaves; 114 genes were upregulated, and 73 genes were down-regulated [60]. Qu et al. compared the gene expression of the hypotoxic discus strain DT-8 and the virus-free strain DT-8 VF and obtained a total of 3110 differentially expressed genes with statistical significance, among which 1741 were up-regulated, and 1369 were down-regulated [61]. This study screened 1746 differentially expressed genes before and after RsPV-BS5 infected *R. solani* strain 06-2-15. There were 1046 upregulated genes and 700 down-regulated genes. Transcriptome data obtained using RNA-seq technology can provide information on fungal response to hypoviral infection and the mechanism through which hypovirulence is induced through the identification of a pool of DEGs [62,63]. In this study, GO and KEGG analyses revealed that the number of DEGs involved in cell-wall-degrading enzyme genes was reduced in the RsPV-BS5-infected strain 06-2-15 and associated with the hypovirulence mediated by RsPV-BS5. The cell wall is the primary barrier of plant resistance to infection with pathogenic fungi. The main components of the cell wall include cellulose, hemicellulose, pectin, etc. Correspondingly, pathogenic fungi can secrete cell wall-degrading enzymes to degrade cell wall components. Reports on PE and PG enzymes showed that the absence of the PE enzyme *Bcpme1* significantly weakened the virulence of *Botrytis cinerea* [64]. The PG-enzyme gene knockout of *Botrytis cinerea* also significantly weakened its pathogenicity [65]. In this study, the expression of PG and PE in host cells decreased after RsPV-BS5 infection, which reduced the plant cell wall degradation by *R. solani*, thus reducing the host’s pathogenicity to plants.

## 5. Conclusions

We have presented molecular evidence showing that RsPV-BS5 isolated from the *R. solani* AG-3 strain BS-5 in Yunnan Province, China, represents a new strain of RhsV-717, named RsPV-BS5. Phylogenetic analysis based on the putative replication protein suggests that RsPV-BS5 may belong to the genus of *Betapartitivirus* in the family of *Partitiviridae*. RsPV-BS5 can spread horizontally and vertically, which reveals its potential for hypovirulence. Transcriptome analysis showed that four cell-wall-degrading-enzymes-related genes were differentially expressed between two isogenic strains, 06-2-15V and 06-2-15. These findings provide new insights into the molecular mechanism of RsPV-BS5 in reducing host pathogenicity.

## Data Availability

The data presented in this study are openly available in GenBank. Nucleotide sequences of RsPV-BS5 under accession number were OK392630 and OK392631.

## Supplementary Materials

The following supporting information can be downloaded at: https://www.mdpi.com/article/10.3390/v15102088/s1, Table S1: Primers used for amplifying the middle fragment sequences, 5′ and 3′ terminal sequences of cDNA from RsPV-BS5 genome in this study; Table S2: Specific primers used for amplifying the genome of RsPV-BS5; Table S3: The information of full-length genomic sequences and their ORF1 and ORF2-encoded proteins aa sequence identity comparison with that of Rhizoctonia solani virus 717 (RshV717) in this study by BLASTn and BLASTp of NCBI; Table S4: Compare the data with the reference genome (*R. solani*); File S1: Gene Ontology and KEGG pathway enrichment analysis; Figure S1: Histogram of GO functional enrichment; Figure S2: Pathway diagram of KEGG metabolism enrichment.
