# Supplementary material for: Horizontal and Vertical Transmission of a Mycovirus Closely Related to the Partitivirus RhsV717 That Confers Hypovirulence in Rhizoctonia solani
Source: Viruses. 2023 Oct 13;15(10):2088. doi: 10.3390/v15102088 (PMC10611285; doi:10.3390/v15102088)
Supplement: Supplementary file 1 [file viruses-15-02088-s001.zip › viruses-2560427-supplementary.pdf]

Table S1. Primers used for amplifying the middle fragment sequences, 5′ and 3′ terminal sequences of cDNA from RsPV-BS5 genome in this study.

Function	Primer name	Primer sequence (5′-3′)
Synthesize genome middle fragment cDNA of RsPV-BS5	B1-64F	ATAATACAAGTCCCGAGC
	B1-64R	TAAAAAAACAGCAAACAA
	B2-64F	CAAAGGCTATCTACCACA
	B2-64R	ATATACATAACGCAAGGA
	B3-64F	ACAAACTCTCAACTCTCG
	B3-64R	TTTATGGTAATCACGGAA
	B4-52F	ACCCAAGCAACAAGAACT
	B4-52R	TGAAGAATCGATTGAACA
	B5-52F	ACTTCACTGCTACTTGGT
	B5-52R	AGGAGATGTTATTTCGTT
	B6-52F	TTACGAACGAACTCTCCT
	B6-52R	TTAATTGCATTTCTATGA
RsPV-BS5 genome terminal specific primers	64-3F1	CGCGCCGAGAACACGTA
	PC2	CCGAATTCCCGGGATCC
	64-3F2	CCACGAGACGACGCTGA
	PC2	CCGAATTCCCGGGATCC
	PC2	CCGAATTCCCGGGATCC
	64-5R1	AAGACGGACGGGGATGG
	PC2	CCGAATTCCCGGGATCC
	64-5R2	CGGCGGCGTTGTTGTAG
	PC2	CCGAATTCCCGGGATCC
	52-5R1	TTGAGAGGCGAAGAGCG
	PC2	CCGAATTCCCGGGATCC
	52-5R2	TAGTACCAGCGGGGCGG
	52-3F1	CGCTACTGCCTCAAACG
	PC2	CCGAATTCCCGGGATCC
	52-3F2	AGCCTACGCCTCATGTGC
	PC2	CCGAATTCCCGGGATCC
PC3-T7 loop primer	PC3-T7	P-GGATCCCGGGAATTCGGTAATACGACTCACTA-TATTTTTATAGTGAGTCGTATTA-NH2

Table S2. Specific primers used for amplifying the genome of RsPV-BS5.

Function	Primer name	Primer sequence (5′-3′)	target fragment
specific primers	64F2	TGAGAAGTGTTTGATCCA	498bp
	64R2	CATAATACAAGTCCCGAG	
	52F1	TACGCAGAGAAACCAACC	266bp
	52R1	TACGCAGAGAAACCAACC	
	BS5CP-F	CCAGTTCTATTCCGTGA	835bp
	BS5CP-R	GCGAAGCATTTCCATTT	

Table S3. The information of full-length genomic sequences and their ORF1 and ORF2-encoded proteins aa sequence identity comparison with that of Rhizoctonia solani virus 717 (RshV717) in this study by BLASTn and BLASTp of NCBI.

			**Nucleinic acidacid sequence comparison**		**Amino acid comparison**		
Virus Name	GenBank Accession Number	Genome size (nt)	query coverage	Nucleinic acidacid sequence identity to RhsV717 RdRp or CP	Amino acid (aa)	query coverage	Amino acid sequence identity to RhsV717 RdRp or CP protein
Rhizoctonia solani partititvirus BS-5 (RsPV-BS5) RdRp	OK392630	2580	92%%	93.82%	730	100%	98.64%
Rhizoctonia solani partititvirus BS-5 (RsPV-BS5) CP	OK392631	2444	90%	93.77%	683	100%	96.34%

Table S4. Compare the data with the reference genome (*R. solani*).

**sample**	**total_reads**	**total_map**	**unique_map**
P06_2_15V_1	46512008	40153252(86.33%)	39362179(84.63%)
P06_2_15V_2	46506662	39875417(85.74%)	39201621(84.29%)
P06_2_15V_3	40661702	34962319(85.98%)	34261712(84.26%)
P06_2_15_1	44920418	38554254(85.83%)	37913867(84.4%)
P06_2_15_2	44169000	38111450(86.29%)	37232173(84.29%)
P06_2_15_3	42904144	37083717(86.43%)	36351539(84.73%)

File S1. Gene Ontology and KEGG pathway enrichment analysis.

After RsPV-BS5 infected strain 06-2-15, the differential genes were mainly involved in protein binding and activation, such as ribonucleoside and nucleoside binding (GO: 0032549 and GO: 0001882) and carboxyl lyase activity (GO: 0016830). In terms of cell composition, differential genes were mainly involved in the formation of cell membranes, such as the overall assembly of cell membranes (GO:0016021), the intrinsic components of cell membranes (GO:0031224), and the components of cell membranes (GO:0044425).

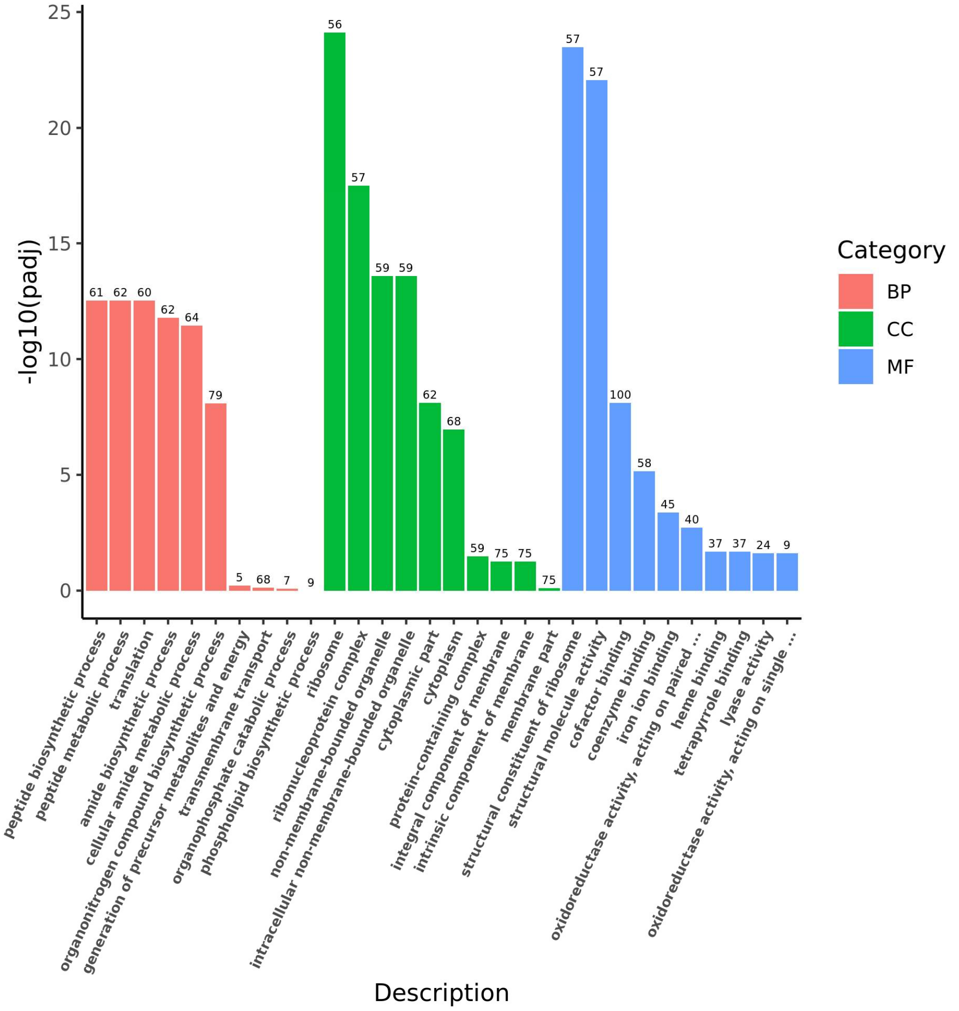

Figure S1. Histogram of GO functional enrichment. Horizontal Coordinate((X-axis)) represents the GO terms, which were categories used to describe the functions of genes and gene products; Vertical Coordinate((Y-axis)) representd the significance level of GO term enrichment, often represented as -log10(padj). The higher the value on the Y-axis, the more significant the enrichment of that GO term in the dataset.

Kyoto Encyclopedia of Genes and Genomes (KEGG) was a comprehensive database integrating genomic, chemical and system function information. For KEGG pathway enrichment, padj less than or equal to 0.05 was taken as the threshold for significant enrichment, and the 20 most significant KEGG pathways were selected in Figure 1-2. The biosynthesis of secondary metabolites (abv01110), ribosomes (abv03010) and amino acids (abv01230) are the top three metabolic pathways. In addition, glycolysis and glucose metabolism synthesis (abv00010), glutathione metabolism (abv00480), alanine, aspartate and glutamate metabolism (abv00250) and tyrosine metabolism (abv00350) were also enriched.

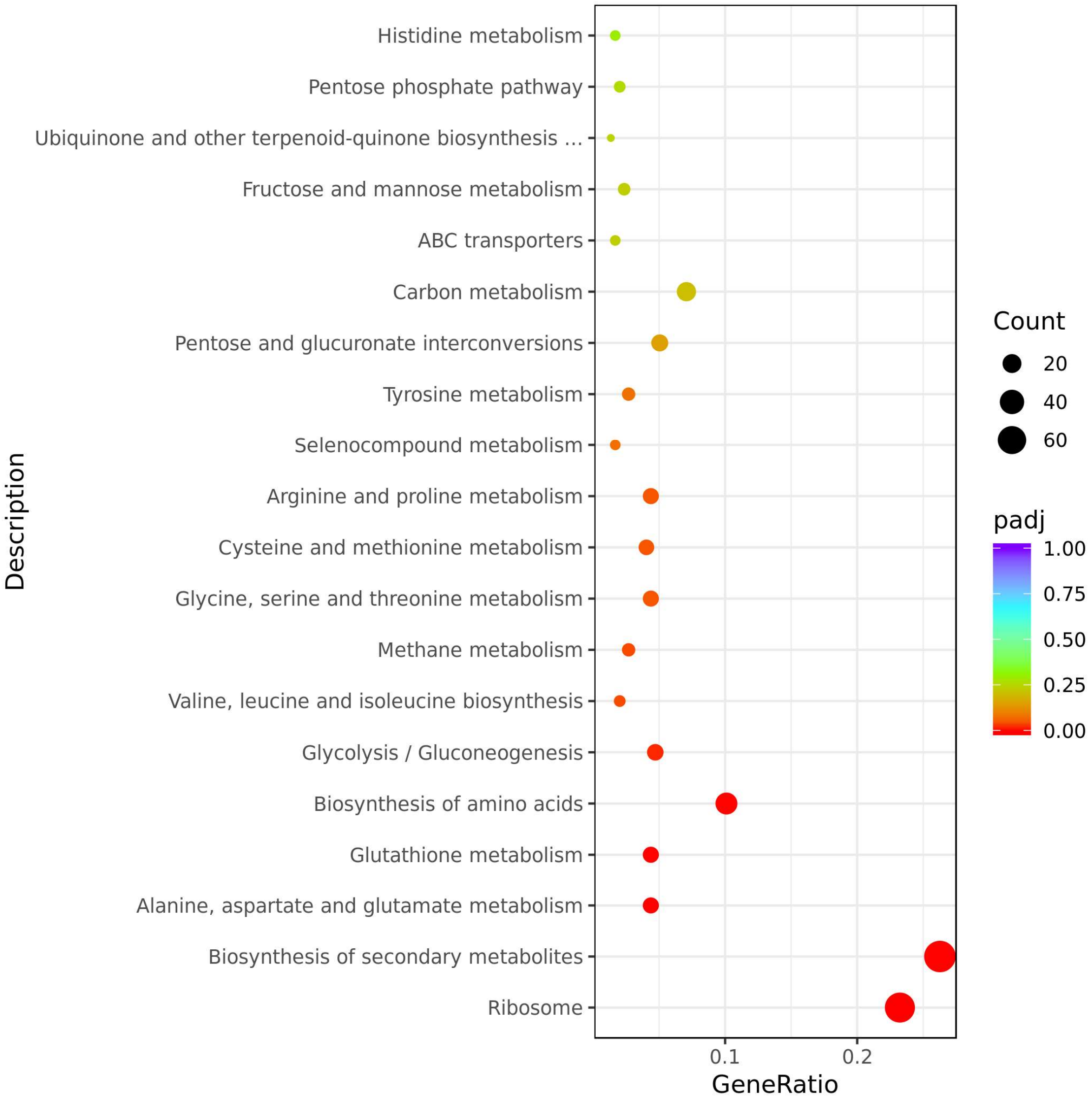

Figure S2. Pathway diagram of KEGG metabolism enrichment. Horizontal coordinate represents the ratio of the number of differential genes that are annotated to a specific KEGG pathway to the total number of differential genes in your dataset; Vertical coordinate represents the KEGG pathways themselves, each KEGG pathway corresponds to a specific biological pathway or network of genes involved in a particular cellular process, such as tyrosine metabolism.
